# Design, synthesis and biological evaluation of a novel colchicine-magnolol hybrid for inhibiting the growth of Lewis lung carcinoma *in Vitro* and *in Vivo*

**DOI:** 10.3389/fchem.2022.1094019

**Published:** 2022-12-13

**Authors:** Zhiyue Li, Shengquan Hu, Liu-Yang Pu, Ziwen Li, Guanbao Zhu, Yongkai Cao, Limin Li, Yucui Ma, Zhanyan Liu, Xinping Li, Guangjie Liu, Keji Chen, Zhengzhi Wu

**Affiliations:** ^1^ Shenzhen Institute of Translational Medicine, Shenzhen Second People’s Hospital, The First Affiliated Hospital of Shenzhen University, Shenzhen, China; ^2^ Shenzhen Institute of Geriatrics, Shenzhen, China; ^3^ Integrated Chinese and Western Medicine Postdoctoral Research Station, Jinan University, Guangzhou, China; ^4^ Guangxi University of Chinese Medicine, Nanning, China; ^5^ Xiyuan Hospital, China Academy of Chinese Medical Sciences, Beijing, China

**Keywords:** colchicine-magnolol hybrid, lewis lung carcinoma cells, extracellular signal-regulated kinase, colchicine binding site, tumor growth inhibition

## Abstract

Colchicine is a bioactive alkaloid originally from Colchicum autumnale and possesses excellent antiproliferative activity. However, colchicine-associated severe toxicity, gastrointestinal side effects in particular, limits its further therapeutic use. In the current study, we thus designed and synthesized a novel hybrid (CMH) by splicing colchicine and magnolol, a multifunctional polyphenol showing favorable gastrointestinal protection. The antitumor activity of CMH in Lewis lung carcinoma (LLC) was then evaluated *in vitro* and *in vivo*. Biologically, CMH inhibited the growth of LLC cells with an IC_50_ of 0.26 μM, 100 times more potently than cisplatin (26.05 μM) did. Meanwhile, the cytotoxicity of CMH was 10-fold lower than that of colchicine in normal human lung cells (BEAS-2B). In C57BL/6 mice xenograft model, CMH (0.5 mg/kg) worked as efficacious as colchicine (0.5 mg/kg) to inhibit tumor growth and 2 times more potently than cisplatin (1 mg/kg). In terms of mortality, 7 out of 10 mice died in colchicine group (0.75 mg/kg), while no death was observed in groups receiving CMH or cisplatin at 0.75 mg/kg. Mechanistic studies using Western blot revealed that CMH dose-dependently suppressed the protein expression of phosphorylated ERK. Molecular docking analysis further indicated that CMH was well fitted in the colchicine binding site of tubulin and formed several hydrogen bonds with tubulin protein. These results enable our novel hybrid CMH as a potential antineoplastic agent with lower toxicity, and provide perquisites for further investigation to confirm the therapeutic potentiality of this novel hybrid.

## 1 Introduction

Lung cancer represents a kind of very common malignant tumor that seriously threatens human life with a persistently high morbidity and mortality rate (Sun and Yan, 2020). Among them, non-small cell lung cancer accounts for the vast majority proportion. Although there are multiple avenues of therapeutic interventions in recent decades, including surgery, chemotherapy and radiation, alone or in combination, the compromised or even destroyed immune system of patients could be often observed in clinical practice (Zhou et al., 2022). Therefore, there is an urgent need to develop an alternative anti-cancer drug or therapy with increased efficacy and reduced toxicity.

Colchicine 1) is a bioactive alkaloid originally isolated from Colchicum autumnale and has long been used as a treatment for gout (Deng et al., 2021). Besides, there is extensive evidence that colchicine has displayed excellent antiproliferative potential in a variety of cancer cell lines against colon, breast, skin melanoma, liver and pancreas (Malik et al., 2022; Song et al., 2022; Wang et al., 2022), and thereby entered into different stages of clinical trials as an anti-cancer agent. Mechanistic studies have revealed that colchicine arrests cell division and kills tumor cells by favorably binding to the colchicine binding site of tubulin and interfering with microtubule formation (Dubey et al., 2017). However, colchicine treatment is always accompanied by serious gastrointestinal side effects (Papageorgiou et al., 2017), including vomiting, diarrhea and abdominal pain nausea, which limits its further clinical application or even causes treatment discontinuation in patients.

Magnolol 2) is a polyphenolic compound from Magnolia officinalis and possesses various pharmacological activities including antioxidation, anti-inflammation and antiangiogenesis (Peng et al., 2022; Xie et al., 2022). Most notably, magnolol has shown favorable gastrointestinal protection in a wide range of experimental paradigms associated with acute gastrointestinal injury and diarrhea (Xia et al., 2019; Lin et al., 2021; Mao et al., 2021).

Since that colchicine exhibits strong antiproliferative ability and magnolol is able to protect against gastrointestinal injury, a common side effect with colchicine treatment, we assume that the drug combination of colchicine and magnolol may provide additive antitumor potential with lower toxicity. Recently, we completed a concise asymmetric synthesis of (–)-colchicine (Pu et al., 2022a) and developed a series of new C-10-modified colchicinoid and evaluated their inhibitory activity on key proteases of 2019-nCoV replication and acute lung injury (Pu et al., 2022b).

**FIGURE 1:**
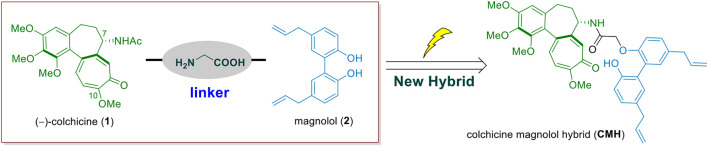
The design of the colchicine-magnolol hybrid (CMH).

While, in order to find new antitumor colchicine analogues with improved activity and lower toxicity, we expect to create a novel C-7-modified colchicinoid with single structure by splicing colchicine and magnolol (Figure 1). In this current study, we thus developed a novel colchicine-magnolol hybrid (CMH) and further evaluated its anti-proliferative potential *in vitro* and *in vivo* as well as the molecular mechanisms involved.

## 2 Results and discussions

### 2.1 Efficient synthesis of novel synthesized a novel hybrid

Small molecules that hit colchicine binding site could exert their efficient biological effects by inhibiting tubulin assembly and suppressing microtubule formation (Lu et al., 2012), numerous modifications of the colchicine chemical structure have been thus made to develop new anti-cancer candidate drug molecules (Gracheva et al., 2020). However, there were few reports about the hybridization of colchicine with other bioactive natural molecules. In this current study, we designed a new type of C-7-modified colchicinoid which was a hybrid of colchicine and magnolol with simple amino acid as the linker.

**SCHEME 1:**
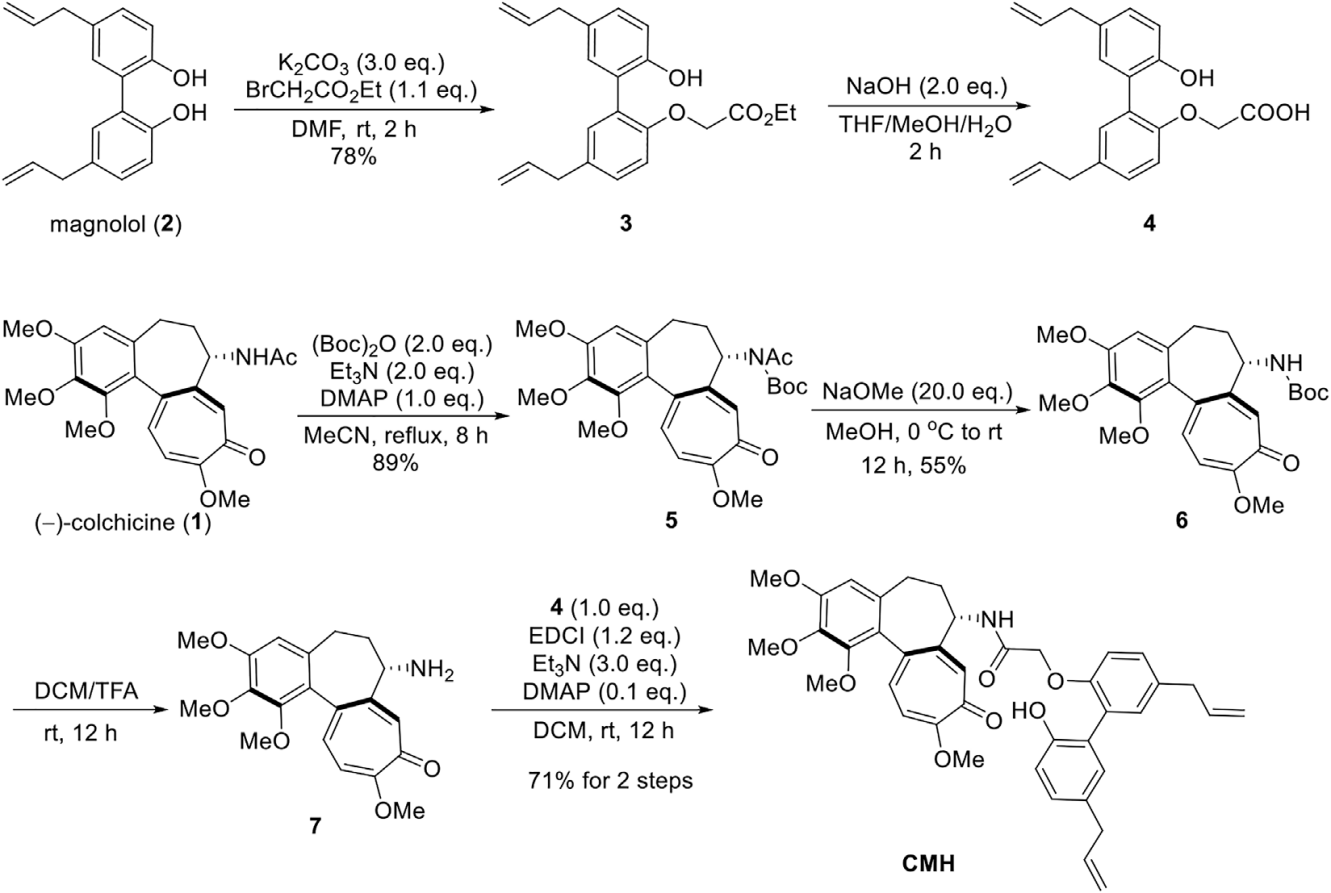
The synthetic route of novel hybrid CMH.

As shown in Scheme 1, the synthesis of CMH was commenced with the S_N_2 reaction of magnolol with ethyl bromoacetate, and compound 3 was isolated in 78% yield. The ester was hydrolyzed with NaOH to generate the acid 4 which was used directly without further purification. In addition, a three-step sequence was employed to remove the -Ac group at C(7) of colchicine (Lagnoux et al., 2005; Yasobu et al., 2011). Furthermore, the amino group was then acylated with compound 4 to give the novel hybrid CMH.

### 2.2 CMH inhibited the proliferation of Lewis Lung Carcinoma cells 100 times more potently than cisplatin did

**FIGURE 2:**
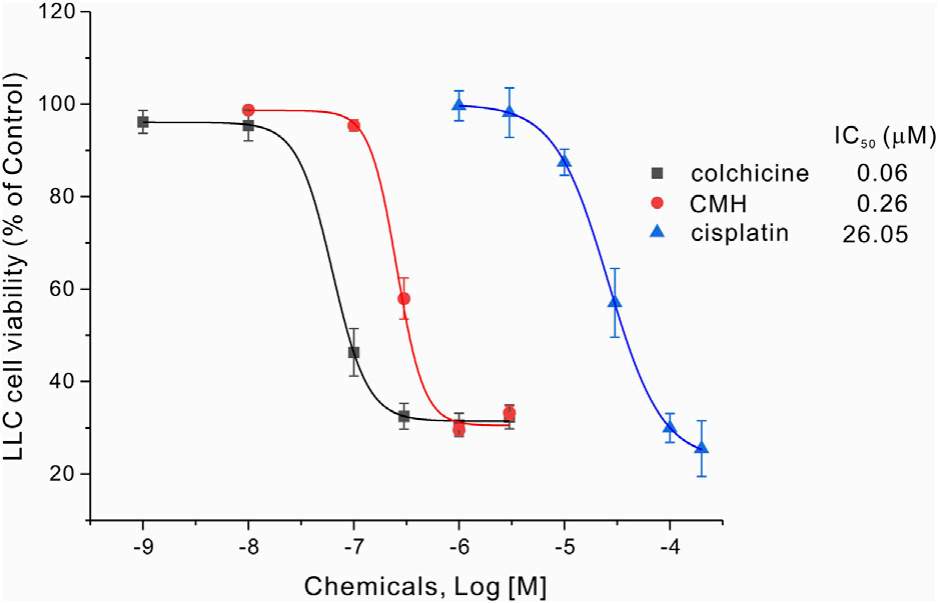
CMH inhibited the proliferation of LLC cells 100 times more potently than cisplatin did. LLC cells were incubated with colchicine, CMH, or cispatin (positive control) for 24 h, and then measured for cell viability.

**FIGURE 3:**
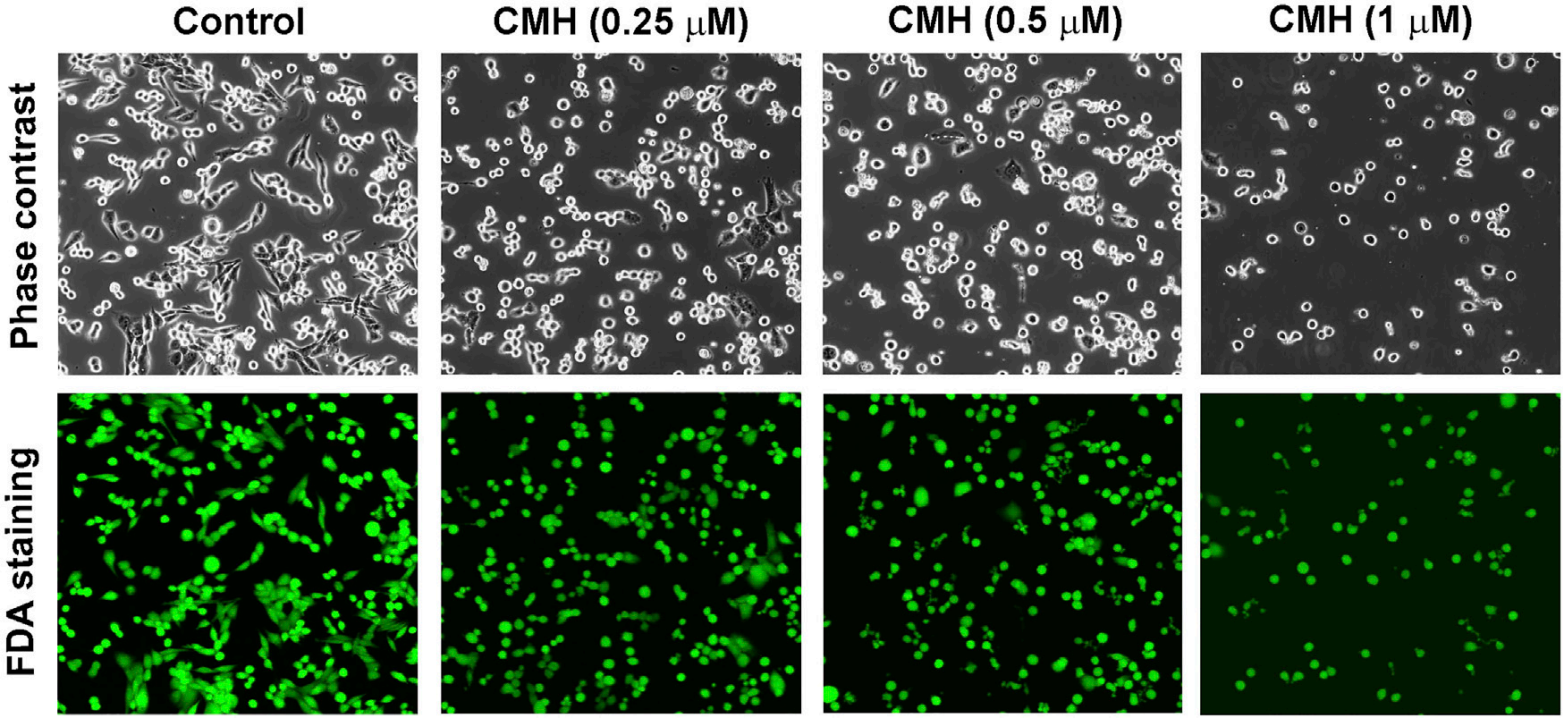
CMH markedly decreased the number of FDA-stained viable cells in a dose-dependent manner. LLC cells were incubated with CMH (0.25 μM, 0.5μM, 1 μM) for 24 h, and then stained with FDA for 5 min, and observed under a confocal laser scanning microscopy.

We then evaluated the antitumor activity of colchicine, CMH and cisplatin (positive control) in Lewis lung carcinoma (LLC) cells. As shown in Figure 2, the cell viability of LLC cells was greatly reduced after 24 h treatment with three compounds. Specifically, the half-maximal inhibitory concentration (IC_50_) of colchicine, CMH and cisplatin against LLC cells was 0.06, 0.26 and 26.05 μM, respectively. It was evident that our novel hybrid CMH inhibited proliferation of LLC cells 100 times more potently than cisplatin did. In consistent with the cell viability results, images from confocal microscopy showed that compared to the control group, a lower density and rounder shape were observed in LLC cells incubated with different concentrations of CMH (Figure 3). Moreover, CMH remarkably decreased the number of FDA-stained viable cells (Figure 3). These results indicated that the proliferation of LLC cells was inhibited to a larger extent by CMH treatment.

### 2.3 CMH caused lower toxicity to BEAS-2B cells compared to colchicine

**FIGURE 4:**
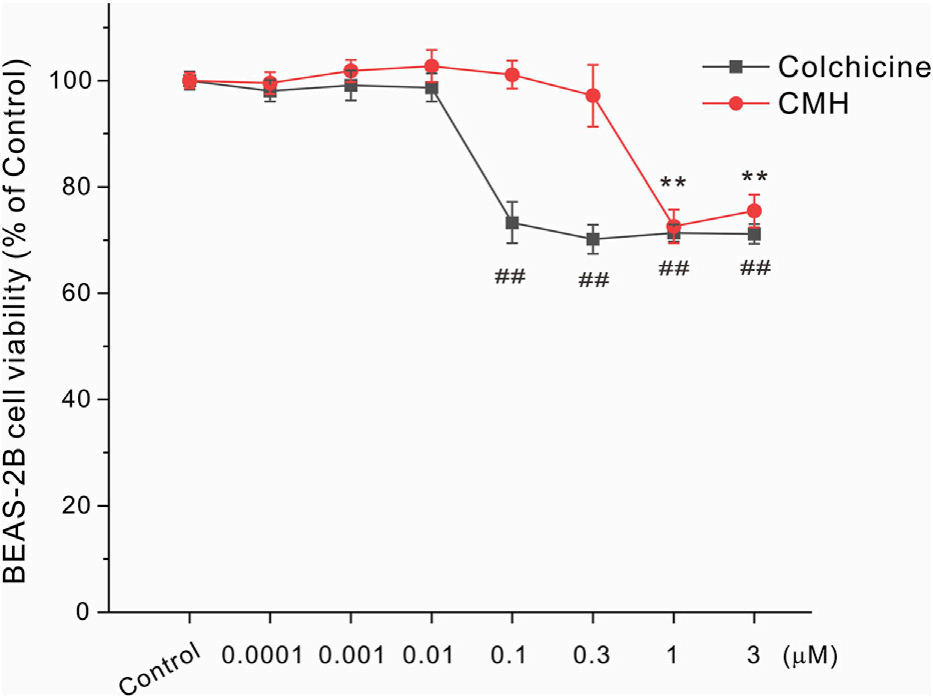
CMH induced lower toxicity in normal BEAS-2B cells compared to its parent molecule colchicine. BEAS-2B cells were incubated with dimethyl sulfoxide (Control), equal concentrations of CMH or colchicine for 24 h, and then examined for cell viability. ^##^, *p* < 0.01, colchicine group *versus* Control; **, CMH group *p* < 0.01 *versus* Control.

Next, we further used normal lung epithelial BEAS-2B cells to evaluate the toxicity of CMH and its parent molecule colchicine. As shown in Figure 4, CMH did not show any toxicity until its concentration reached 1 μM. Specifically, 24 h exposure of BEAS-2B cells to CMH at 1 μM decreased cell viability from (100.00 ± 1.48) % to (72.59 ± 3.17) %. In comparison, colchicine began to induce toxicity at the concentration of 0.1 μM, colchicine at 0.1 μM decreased cell viability to (73.27 ± 3.89) %. These results indicated the cytotoxicity of CMH might be 10-fold lower than that of colchicine in normal human lung cells.

### 2.4 CMH at high concentration inhibited the activity of GSK3β

**FIGURE 5:**
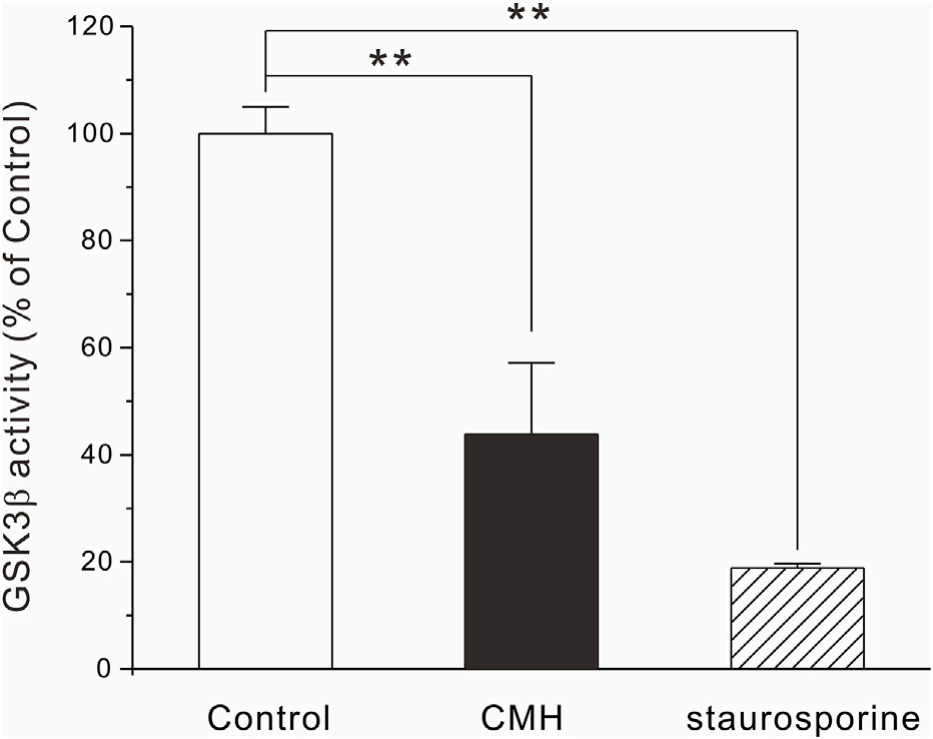
CMH at high concentration inhibited GSK3β activity. 1 ng GSK3β, 0.2 μg/μl substrate, 25 μM ATP and CMH (final concentration, 100 μM) or staurosporine (0.1 μM, positive control) were incubated in each well of 384-well plate for 60 min. ADP-Glo™ and kinase detection reagent was introduced successively into the well and luminescence was read. **, *p* < 0.01 *versus* Control.

Glycogen synthase kinase-3β (GSK3β) is a serine-threonine kinase that is responsible for promoting cancer cell survival, growth and proliferation (Domoto et al., 2020). It has been well documented that aberrant GSK3β activity is firmly associated with multiple tumor-related diseases and that GSK3β is generally accepted as a potential anti-tumor target (Bai et al., 2022; Fan et al., 2022; You et al., 2022). In light of this, we extended our effort to test the possibility that CMH may provide antiproliferative capacity through the direct inhibition of GSK3β enzyme activity. As shown in Figure 5, CMH at 100 μM decreased enzyme activity to approximately 40% of control, while failed to inhibit GSK3β at concentrations below 100 μM (data not shown). Since CMH showed toxicity in normal lung epithelial BEAS-2B cells when its concentration exceeded 1 μM, we speculate that GSK3β may not be the critical mechanism underlying CMH-mediated antitumor efficacy and some other potential targets are expected to be explored.

### 2.5 CMH downregulated the protein expression of phospho-ERK in LLC cells

There is extensive evidence that the phosphorylation of mitogen-activated protein kinases (MAPKs), extracellular signal-regulated kinase (ERK) subtype in particular, is closely associated with the growth and proliferation of tumor cells in cellular and animal experimental paradigms (Yang et al., 2022; Yu et al., 2022). Specifically, the increase of phospho-ERK level was usually observed in cancer cell lines such as LLC cells, particularly those treated with pro-tumorogenic compounds (Stoyanov et al., 2012), bio-molecules that could down-regulate ERK phosphorylation may thereby offer effective anti-tumor efficacy (Shi et al., 2022; Yuan et al., 2022).

**FIGURE 6:**
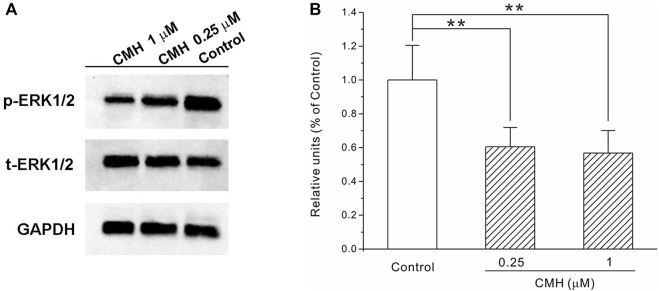
CMH strongly down-regulated the protein expression of phospho-ERK in LLC cells. LLC cells were incubated with CMH (0.25 μM, 1 μM) for 24 h, and then measured for protein expression. **, *p* < 0.01 *versus* Control. **(A)** Representative bands of p-ERK, t-ERK and GAPDH. **(B)** The statistical analysis.

In our cell system, the protein level of phospho-ERK (p-ERK) was evaluated using Western blot and the results in Figure 6 showed that CMH at 0.25 μM and 1 μM declined this phosphorylated protein to (0.61 ± 0.11) and (0.57 ± 0.13), respectively, compared to the control group (1.00 ± 0.20). This phenomenon was consistent with earlier findings where this phosphorylated protein level in LLC cells greatly declined in the presence of anti-tumorogenic chemicals (Kim et al., 2007; Xie et al., 2018) and indicated that the inhibition of p-ERK may be a critical mechanism that underlied the anti-tumor effects of CMH.

### 2.6 CMH was well fitted in the colchicine binding site of tubulin and formed several hydrogen bonds with tubulin

**FIGURE 7:**
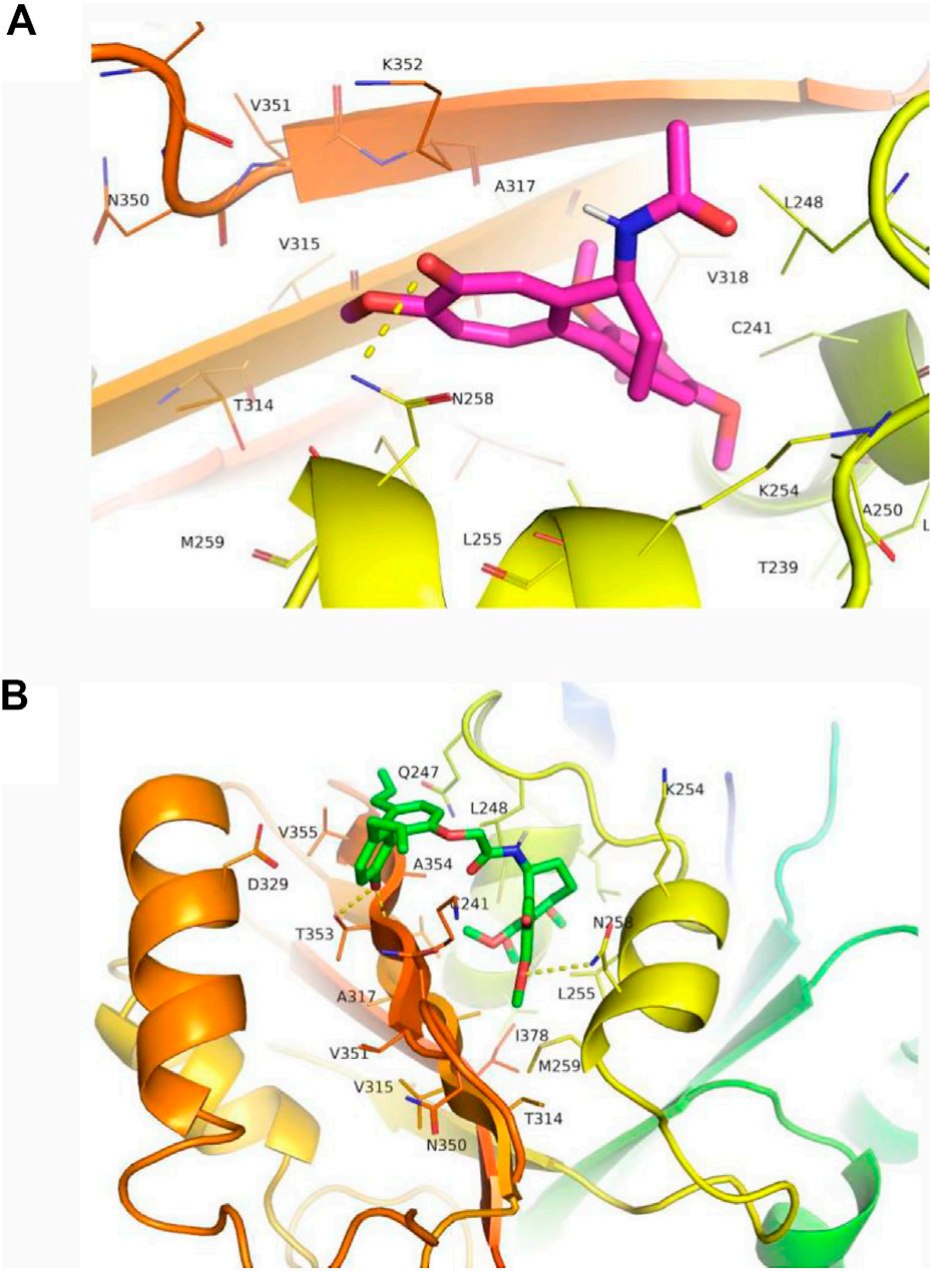
CMH interacted with tubulin at the colchicine binding site. The interaction between colchicine **(A)** or CMH **(B)** with tubulin. CMH was well fitted in the colchicine binding site of tubulin with binding energies of -8.04 kcal/mol, in comparison to the colchicine ligand (-5.93 kcal/mol).

Microtubules, which maintain the shape of the cell through the dynamic assembly of tubulin heterodimers, are generally accepted as an attractive target for the development of anti-cancer drugs (Andres et al., 2022). Specifically, colchicine binding site agents bind to colchicine binding domain and prevent the polymerization of tubulin proteins, thereby destabilize microtubules and provide antitumor potential (Wang et al., 2016; Sueth-Santiago et al., 2017). We then tested the possibility that our novel hybrid CMH could occupy colchicine binding site. Results from docking studies of compounds with tubulin protein (PDB entry: 1SA0) showed that both colchicine and CMH were well fitted in the colchicine binding site of tubulin. Specifically, colchicine molecule formed a hydrogen bond to Asn258 of tubulin protein with an estimated binding free energy of -5.93 kcal/mol, an observation consistent with an earlier study (colchicine docking score: −5.5 kcal/mol) (Dwivedi et al., 2022). In contrast, the best-docked conformation of CMH in the tubulin showed that the methoxy and the phenolic hydroxyl group of this ligand from the colchicine fragment and B fragment interacted with Asn258 and Thr353 through several hydrogen bonds with an estimated binding free energy of -8.04 kcal/mol (Figure 7). These results suggested that our novel CMH could be served as an effective colchicine binding site inhibitor.

In this docking system, CMH showed better docking score than the standard inhibitor colchicine, on the other hand, CMH (IC_50_ = 0.26 μM) displayed anti-proliferative potential 4 times less potently than colchicine (IC_50_ = 0.06 μM, Figure 2). This discrepancy could be explained by the existence of some other possible targets, such as taxane, vinca, laulimalide binding domains of tubulin, that CMH or colchicine may hit. Such interesting topics will be further investigated in our future projects.

### 2.7 CMH exhibited robust suppression of tumor growth in C57BL/6 mice xenografted with LLC cells

**FIGURE 8:**
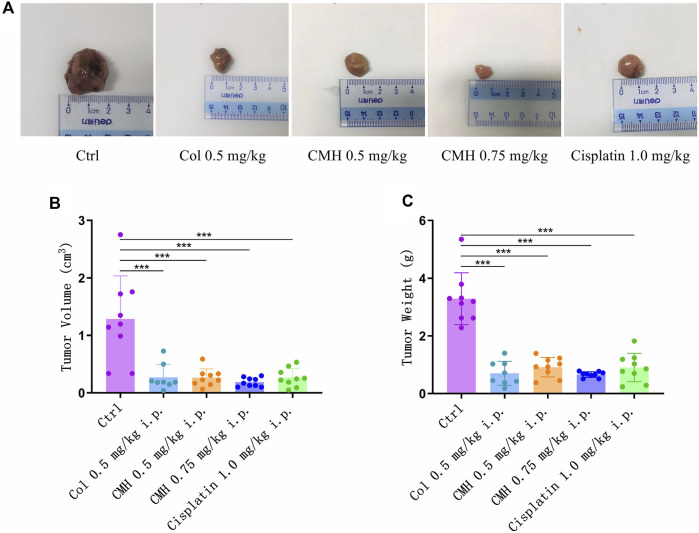
Effects of colchicine (Col, 0.5 mg/kg), CMH- (0.5 and 0.75 mg/kg) and cisplatin (1.0 mg/kg) on the tumor volume and weight in the xenografted mice during the entire experimental period. **(A)** Representative macroscopic view of LLC tumors in different groups. **(B)** Quantitative analysis of tumor volume. **(C)** Quantitative analysis of tumor weight. ^***^ *p* < 0.001 *versus* control group.

Finally, the antitumor ability of CMH was verified in C57BL/6 mice xenograft model. As shown in Figure 8, CMH exhibited promising antitumor efficacy, with a tumor growth inhibition (TGI) of 79.37% and 85.44% at the dosages of 0.5 mg/kg and 0.75 mg/kg, respectively. No mortality was observed in the group treated with CMH. For a reference, cisplatin (positive control) at 1.0 mg/kg inhibited tumor growth by 79.22% and colchicine at 0.5 mg/kg inhibited tumor growth by 78.81%. However, colchicine treatment was accompanied with a high risk of mortality. It was obvious that 7 out of 10 mice (colchicine group, 0.75 mg/kg) and one out of 9 mice (colchicine group, 0.5 mg/kg) were dead during 10 days of drug treatment (Table 1). These findings taken together suggested that CMH inhibited tumor growth 2 times more potently than cisplatin, and that CMH displayed antitumor capacity with a lower mortality and an efficacy comparable or even superior to colchicine.

**TABLE 1:** Inhibitory effects of compounds on tumor weights and tumor volume in the C57BL/6 mice xenografted LLC cells.

Groups	Avg. tumor weights (g)	Avg. tumor volume (cm^3^)	%TGI	Mortality
Control	3.29 ± 0.90	1.29 ± 0.75	0	0/9
Col (0.5 mg/kg)	0.70 ± 0.42^a^	0.27 ± 0.23^a^	78.81	1/9
Col (0.75 mg/kg)^b^	—	—	—	7/9
CMH (0.5 mg/kg)	0.92 ± 0.34^a^	0.27 ± 0.15^a^	79.37	0/9
CMH (0.75 mg/kg)	0.66 ± 0.10^a^	0.19 ± 0.079^a^	85.44	0/9
Cisplatin (1 mg/kg)	0.90 ± 0.49^a^	0.27 ± 0.16^a^	79.22	0/9

^a^ *p* < 0.001 versus the Control group.

^b^ Colchicine was extremely toxic at 0.75 mg/kg, %TGI was thus not determined.

## 3 Conclusion

In conclusion, we herein designed and synthesized a novel hybrid CMH by splicing colchicine and magnolol. CMH exhibited excellent antiproliferative effects in LLC cells (IC_50_ = 0.26 μM) and robustly suppressed tumor growth in C57BL/6 mice xenograft model. Meanwhile, CMH showed lower toxicity in normal human lung BEAS-2B cells and in mice when compared to its parent molecule colchicine. Mechanistic studies revealed that CMH provided its antitumor potential mainly through suppressing ERK signaling pathway and occupying colchicine binding site of tubulin concurrently. These results identify our novel hybrid CMH as a potential antineoplastic agent with lower toxicity, and provide perquisites for further investigation to confirm the therapeutic potentiality of this novel hybrid.

## Data Availability

The raw data supporting the conclusions of this article will be made available by the authors, without undue reservation.
