# Supplementary material for: Design, synthesis and biological evaluation of a novel colchicine-magnolol hybrid for inhibiting the growth of Lewis lung carcinoma in Vitro and in Vivo
Source: Front Chem. 2022 Dec 13;10:1094019. doi: 10.3389/fchem.2022.1094019 (PMC9792613; doi:10.3389/fchem.2022.1094019)
Supplement: Supplementary file 1 [file DataSheet1.docx]

Supporting Information For

**Design, Synthesis and Biological Evaluation of a Novel Colchicine-Magnolol Hybrid for Inhibiting the Growth of Lewis Lung Carcinoma *in Vitro* and *in Vivo***

**Zhiyue Li^1,2,†^, Shengquan Hu^1,2,†^, Liu-Yang Pu^1,2,3,†^, Ziwen Li^1^, Guanbao Zhu^1,5^, Yongkai Cao^1^, Limin Li^1^, Yucui Ma^1^*,* Zhanyan Liu^1^, Xinping Li^1^, Guangjie Liu^1^, Keji Chen^4*^，Zhengzhi Wu^1,2,*^**

^1^Shenzhen Institute of Translational Medicine, Shenzhen Second People’s Hospital, The First Affiliated Hospital of Shenzhen University, Shenzhen, China.

^2^Shenzhen Institute of Geriatrics, Shenzhen, China.

^3^Integrated Chinese and Western Medicine Postdoctoral Research Station, Jinan University, Guangzhou, China.

^4^Xiyuan Hospital, China Academy of Chinese Medical Sciences, Beijing, China

^5^Guangxi University of Chinese Medicine, Nanning, China.

***Correspondence:**Zhengzhi Wu, Foreign Academician, the National Academy of Engineering Sciences, Ukraine. Email: [szwzz001@163.com](mailto:szwzz001@163.com).

Keji Chen, Academician, Chinese Academy of Sciences, China. Email: kjchenvip@163.com

†These authors have contributed equally to this work and share first authorship.

**Table of Contents**

1. **Organic Synthesis Experiments…...……………………………………...…………………......S2**
2. **Biological Experiments…………………………………………………………………….…......S4**
3. **NMR Spectra…………………………………………………………………….……………......S7**

**References……………………………………………………………………….…………………......S9**

**1.** **Organic Synthesis Experiments**

**1.1 General Information**

All reactions and manipulations which are sensitive to moisture or air were performed under inert atmosphere of nitrogen. Petroleum ether (PE) refers to the fraction boiling in the 60–90 °C range. Anhydrous MeCN and MeOH were distilled from calcium hydride. NMR spectra were recorded on a Bruker AV 400 spectrometer at 400 MHz (^1^H NMR), 101 MHz (^13^C NMR). Chemical shifts were reported in ppm relative to internal TMS for ^1^H NMR data, deuterated solvent for ^13^C NMR data, respectively. Data are presented in the following space: chemical shift, multiplicity, coupling constant in hertz (Hz), and signal area integration in natural numbers. Melting points were measured on a RY-I apparatus and uncorrected. Optical rotations were determined using a WXG-4 circular polarimeter. High-resolution mass spectra (HRMS) were obtained by electrospray ionization (ESI) on Bruker Daltonics 7 T mass spectrometer in positive ion mode.

**1.2 Synthesis of CMH**

**Synthesis of compound 3**

To the solution of magnolol (3.0 g, 11.3 mmol) in DMF (30 mL) was added K_2_CO_3_ (4.7 g, 33.9 mmol, 3.0 eq.) and BrCH_2_CO_2_Et (1.4 mL, 12.4 mmol, 1.1 eq.). The mixture was stirred at room temperature for 2 h. Then 1 N HCl was added to quench the reaction. The mixture was extracted with ethyl acetate (4 × 30 mL). The combined organic phase was dried over anhydrous Na_2_SO_4_ and concentrated in vacuo. The residue was chromatographed on silica-gel column with petroleum ether/ethyl acetate (10:1) to give the product **3** (3.1 g, 78% yield) as a colorless oil. ^1^H NMR (400 MHz, Chloroform-*d*) δ 7.19 – 7.08 (m, 3H), 7.03 (d, *J* = 2.3 Hz, 1H), 6.96 (d, *J* = 8.1 Hz, 1H), 6.79 – 6.72 (m, 1H), 6.68 (s, 1H), 5.98 (dtt, *J* = 16.7, 9.8, 6.8 Hz, 2H), 5.19 – 5.01 (m, 4H), 4.73 (s, 2H), 4.27 (q, *J* = 7.2 Hz, 2H), 3.47 – 3.30 (m, 4H), 1.29 (t, *J* = 7.1 Hz, 3H); ^13^C NMR (101 MHz, Chloroform-*d*) δ 169.85, 152.44, 152.12, 138.04, 137.48, 134.44, 132.34, 131.18, 129.59, 129.19, 127.67, 125.90, 117.68, 116.15, 115.70, 110.64, 64.53, 62.25, 39.61, 39.48, 14.26; HRMS (*m/z*): calcd for C_22_H_24_NaO_4_ ([M + Na]^+^) 375.1567, found 375.1564.

**Synthesis of compound 4**

To the solution of compound **3** (3.1 g, 8.8 mmol) in THF (20 mL) and MeOH (10 mL), was added aq. NaOH (704 mg, 17.6 mmol, 2.0 eq., in 10 mL H_2_O). The mixture was stirred at room temperature for 2 h. Then 1N HCl was added to neutralize the solution. The mixture was concentrated in vacuo, and the residue was extracted with DCM (3 × 30 mL). The combined organic phase was dried over anhydrous Na_2_SO_4_ and concentrated in vacuo to yield the compound **4** which was used without purification.

**Synthesis of compound 5** (Lagnoux et al., 2005)

To the solution of (–)-colchicine (1.5 g, 3.76 mmol) in MeCN (30 mL) was added (Boc)_2_O (1.7 mL, 7.5 mmol, 2.0 eq.) and Et_3_N (1.0 mL, 7.5 mmol, 2.0 eq.) and DMAP (459 mg, 3.76 mmol, 1.0 eq.). The solution was refluxed for 8 h. The contents were loaded directly on silica and the product was separated by column chromatography with ethyl acetate to give the product **5** (1.7 g, 89% yield). ^1^H NMR (400 MHz, CDCl_3_) δ: 7.52 (s, 1H), 7.15 (dd, *J* = 10.6, 1.5 Hz, 1H), 6.71 (dd, *J* = 10.9, 1.6 Hz, 1H), 6.46 (s, 1H), 5.07 (dd, *J* = 12.4, 6.0 Hz, 1H), 3.90 (s, 3H), 3.86 (s, 3H), 3.82 (s, 3H), 3.58 (s, 3H), 2.66–2.47 (m, 2H), 2.45–2.37 (m, 1H), 2.21 (s, 3H), 1.94–1.85 (m, 1H), 1.49 (s, 9H).

**Synthesis of compound 6** (Lagnoux et al., 2005)

To the solution of compound **5** (1.2 g, 2.4 mmol) in MeOH (12 mL) was added NaOMe (1.8 mL, 9.6 mmol, 4.0 eq., 5.4 M in MeOH) at 0 °C. Then the solution was warmed to room temperature naturally and stirred for 12 h. The contents were loaded directly on silica gel and the product was separated by column chromatography with ethyl acetate/methanol (10:1) to give the product **6** (604 mg, 55% yield). ^1^H NMR (400 MHz, CDCl_3_) δ: 7.53 (s, 1H), 7.28 (d, *J* = 10.8 Hz, 1H), 6.82 (d, *J* = 10.8 Hz, 1H), 6.53 (s, 1H), 5.07 (d, *J* = 7.7 Hz, 1H), 4.41 (dt, *J* = 11.5, 6.9 Hz, 1H), 4.00 (s, 3H), 3.94 (s, 4H), 3.90 (s, 3H), 3.66 (s, 3H), 2.56–2.48 (m, 1H), 2.43–2.35 (m, 1H), 2.32–2.22 (m, 1H), 1.73–1.66 (m, 1H), 1.37 (s, 9H).

**Synthesis of compound CMH**

To the solution of compound **6** (604 mg, 1.3 mmol) in DCM (10 mL) was added TFA (2.5 mL). The mixture was stirred at room temperature for 12 h until the reaction was completed. The solution was directly concentrated in vacuo. The residue was dissolved in DCM (20 mL). Then to the solution was added compound **4**, EDCI (299 mg, 1.56 mmol, 1.2 eq.), Et_3_N (541 μL, 3.9 mmol, 3.0 eq.) and DMAP (16 mg, 0.13 mmol, 0.1 eq.). The mixture was stirred at room temperature for 12 h. The reaction solution was quenched with H_2_O (20 mL) and extracted with DCM (3 × 20 mL). The combined organic phase was dried over anhydrous Na_2_SO_4_, and concentrated in vacuo. The residue was chromatographed on silica-gel column by gradient eluted with petroleum ether/ethyl acetate (1:1) to 100% ethyl acetate to give the product **CMH** (613 mg, 71% yield) as a light-yellow solid: mp 128–130 °C; *R*_f_ 0.65 (ethyl acetate); [α] –23.0 (*c* 1.3, CH_2_Cl_2_); ^1^H NMR (400 MHz, CDCl_3_) δ: 9.28 (s, 1H), 7.44 (s, 1H), 7.38 (d, *J* = 10.9 Hz, 1H), 7.20–7.12 (m, 2H), 7.04 (d, *J* = 2.2 Hz, 1H), 7.01–6.87 (m, 3H), 6.83–6.79 (m, 1H), 6.51 (s, 1H), 6.05–5.92 (m, 2H), 5.18–4.99 (m, 4H), 4.67 (ddd, *J* = 11.5, 8.6, 6.4 Hz, 1H), 4.49 (d, *J* = 14.4 Hz, 1H), 4.42 (d, *J* = 14.5 Hz, 1H), 4.03 (s, 3H), 3.93 (s, 3H), 3.90 (s, 3H), 3.62 (s, 3H), 3.38 (dd, *J* = 20.8, 6.8 Hz, 4H), 2.47 (dd, *J* = 13.6, 6.4 Hz, 1H), 2.33 (td, *J* = 13.2, 7.0 Hz, 1H), 2.08–1.98 (m, 1H), 1.34–1.27 (m, 1H); ^13^C NMR (101 MHz, CDCl_3_) δ: 179.44, 167.90, 164.01, 153.65, 152.85, 152.54, 151.57, 151.09, 141.63, 138.00, 137.47, 136.95, 135.99, 134.13, 133.68, 131.57, 131.04, 130.25, 128.79, 128.56, 125.90, 125.15, 115.79, 115.53, 115.31, 113.62, 111.91, 107.37, 67.26, 61.44, 61.31, 56.42, 56.08, 50.75, 39.38, 39.33, 36.71, 29.73; HRMS (*m/z*): calcd for C_40_H_41_NNaO_8_ ([M + Na]^+^) 686.2724, found 686.2714.

**2. Biological Experiments**

**Materials**

High-glucose Dulbecco’s modified Eagle’s medium (DMEM) and fetal bovine serum (FBS) were purchase from Gibco (Grand Island, NY, USA). Penicillin-Streptomycin Solution (100×). Cell counting Kit-8 (CCK-8) was from Beijing Fluorescence Biotechnology Co. Ltd (Beijing, China). Cisplatin was from MCE (Medchem Express, USA). Fluorescein Diacetate (FDA) was from Sigma (St. Louis, MO, USA). Antibodies against phospho-ERK, ERK and GAPDH were from Cell Signaling Technology (Danvers, MA, USA).

**Cell culture**

Murine Lewis Lung Carcinoma (LLC) cells were obtained from iCell Bioscience Inc. (Shanghai, China), normal lung epithelial cells (BEAS-2B) were from Procell (Wuhan, China). Both cell lines were cultured in DMEM that contained 10% FBS and Penicillin-Streptomycin (1×) in a 37℃ CO_2_ incubator.

**Cytotoxicity assay**

LLC cells and BEAS-2B cells were seeded in 96-well plates at a density of 6×10^3^ cells/well and 3×10^4^ cells/well, respectively. 24 h later, cells were incubated with increasing concentrations of CMH, colchicine or cisplatin for another 24 h. After that, the medium was removed and CCK-8 reagent was added to each well for 2 h. Absorbance values at 450 nm wavelength were immediately read on a microplate reader (SpectraMax i3x, Molecular Devices, USA).

**FDA staining**

FDA is a fluorescent probe used to stain viable cells based on the principle that FDA could be served as a substrate for active enzymes inside the cells. FDA staining assay was performed as we previously reported (Hu et al., 2018). In our cell system, LLC cells treated with or without CMH were incubated with FDA solution at a final concentration of 10 μg/mL for 5 min, and then observed using a confocal laser scanning microscopy (FLUOVIEW FV1Oi, Olympus).

**GSK3β activity assay**

GSK3β activity was evaluated using the ADP-Glo kinase assay + GSK3β kinase enzyme system (Promega, V9371). In brief, 1 ng GSK3β, 0.2 μg/μL substrate, 25 μM ATP and CMH or staurosporine (positive control) were incubated in 384-well white low volume plate at room temperature for 60 min. Thereafter, ADP-Glo^TM^ reagent was added into each well and further incubated for 40 min. This enzymatic reaction was stopped by introducing kinase detection reagent. Luminescence was read on the microplate reader.

**Western blot**

Western blot analysis for evaluating the signaling pathways was carried out as we described (Hu et al., 2020). Briefly, the cultured medium was removed and LLC cells in each well of 6-well plates were incubated for 15 min with 70 μL lysis buffer. The extracted protein was then quantitated, fully denatured and loaded onto a 12 % SDS-PAGE gel. After transfer of the proteins onto PVDF membranes, the blot was probed with anti-phospho-ERK1/2, anti-ERK1/2 and GAPDH (internal control) antibodies, followed by horseradish peroxidase-conjugated secondary antibodies. The signal detection was done using Pierce ^TM^ ECL Western Blotting Substrate.

**Molecular docking and data processing**

The crystal structure of tubulin (PDB ID: 1SA0) was obtained from the RSCB PDB database (http://www.rcsb.org/) and saved as a PDB format file. The chains A, C, and D were deleted and the remaining chain B was added hydrogens and charges by Discovery Studio 4.1. The structures of the proposed ligands were drawn through ChemDraw 15.0 and then converted to 3D structure following energy minimization by Chem 3D 15.0. The docking was conducted on the LeDock program (<http://lephar.com>) (Cao et al., 2020). The refined protein (tubulin) was imported into the LePro module of LeDock to generate docking parameters. The LeDockGUI plugin on the PyMOL (http://www.pymol.org/) was used to generate a binding pocket (109.7 128.2, 79.6 99.8, -2.1 14.0). Subsequently, the ligands (colchicine and CMH) were docked into the binding site based on a genetic algorithm. For each compound, 50 poses were generated and clustered. The docking results were visualized by PyMOL and binding affinities were predicted by LeDock.

**Mouse xenograft models and drug treatment**

All protocols were approved by the local Animal Care Committee at the Fist Affiliated Hospital of Shenzhen University. Fifty-four male, 8-week-old, 20-25 g C57BL/6 mice (Vital River, Shenzhen, China) were maintained in the central animal laboratory for at least 1 week. To establish tumors, live LLC cells (1×10^6^) re-suspended in PBS were subcutaneously inoculated into the left flank of each mouse. 7 days later, when tumors had reached approximately 5 mm in diameter, the mice were randomly divided into five equal groups and treated intraperitoneally with different compounds for 10 days: 1) Control (saline); 2) Colchicine (0.50 mg/kg) ; 3) Colchicine (0.75 mg/kg) ;4) CMH (0.50 mg/kg); 5) CMH (0.75 mg/kg) ; 6) Cisplatin (1 mg/kg).

**Tumor weight and tumor growth inhibition calculation**

Tumor size and weight were measured using calipers and scales on day 13, and the volume (V) was calculated according to the formula V=A×B^2^×0.52, where A is the largest superficial diameter and B is the smallest superficial diameter. The percentage of tumor growth inhibition (%TGI) was calculated as follows: %TGI=[1-(T/C)] × 100. T and C stand for the tumor volume in treatment group and control group, respectively.

**Statistical analysis.**

Experimental data were presented as mean ± S.D. Statistical analysis was performed using GraphPad Prism 8.0 software. Bonferroni’s multiple comparison test after one-way ANOVA analysis was employed. Significance was considered as follows: ^***^*p* < 0.001, ^**^*p* < 0.01, ^*^*p* < 0.05.

1. **NMR Spectra**

**Reference**

Cao, Y., Min, C., Acharya, S., Kim, K. M., Cheon, S. H. (2016). Design, synthesis and evaluation of bitopic arylpiperazinephenyl-1,2,4-oxadiazoles as preferential dopamine D3 receptor ligands. *Bioorg. Med. Chem*. 24, 191–200. doi: 10.1016/j.bmc.2015.12.002.

Hu, S., Hu, H., Mak, S., Cui, G., Lee, M., Shan, L., Wang, Y., Lin, H., Zhang, Z., Han, Y. (2018). A Novel Tetramethylpyrazine Derivative Prophylactically Protects against Glutamate-Induced Excitotoxicity in Primary Neurons through the Blockage of N-Methyl-D-aspartate Receptor. *Front. Pharmacol.* 9, 73. doi: 10.3389/fphar.2018.00073.

Hu, S., Xian, Y., Fan, Y., Mak, S., Wang, J., Tang, J., Pang, Y., Pi, R., Tsim, K. W., Liu, F., Lin, Z., Han, Y. (2020). Significant combination of Aβ aggregation inhibitory and neuroprotective properties in silico, in vitro and in vivo by bis(propyl)-cognitin, a multifunctional anti-Alzheimer's agent. *Eur. J. Pharmacol.* 876, 173065. doi: 10.1016/j.ejphar.2020.173065.

Lagnoux, D., Darbre, T., Schmitz, M. L., Reymond, J. L. (2005) Inhibition of mitosis by glycopeptide dendrimer conjugates of colchicine. *Chem. Eur. J*. 11, 3941. doi: 10.1002/chem.200401294.
